# Insights in AAV-mediated antigen-specific immunity and a strategy for AAV vaccine dose reduction through AAV-extracellular vesicle association

**DOI:** 10.1016/j.omtm.2024.101358

**Published:** 2024-10-18

**Authors:** Ester Molina, Marcos Tejero, Ozgun Firat Duzenli, Hisae Kuoch, Colin Caine, Karina Krotova, Michael Paulaitis, George Aslanidi

**Affiliations:** 1 The Hormel Institute, University of Minnesota, Austin, MN 55912, USA; 2 Department of Ophthalmology, Center for Nanomedicine at the Wilmer Eye Institute, Johns Hopkins University School of Medicine, Baltimore, MD 21287, USA; 3 Masonic Cancer Center, University of Minnesota, Minneapolis, MN 5455, USA

**Keywords:** adeno-associated virus, dendritic cell, extracellular vesicles, cancer vaccine, antigen-specific T cell, protective immune response

## Abstract

We previously showed therapeutic advantages of using a capsid-modified and encoded antigen-optimized AAV-based cancer vaccine to initiate strong antigen-specific immune responses and increase survival in a syngeneic mouse model of melanoma. In this study, we further explore AAV vaccine dose reduction and possible mechanisms of the immune response. Immunization with extracellular vesicle (EV)-associated AAV6-S663V encoded ovalbumin (OVA) or tyrosinase-related protein 1 (TRP-1) induced significantly higher levels of antigen-specific CD8^+^ T cells compared with standard AAV in mice. Importantly, a higher number of specific CD8^+^ T cells was achieved with EV-AAV several logs lower than optAAV-based doses. EV-optAAV-OVA was used in a dose 100 times lower, and EV-optTRP-1 10 times lower than optOVA and optTRP-1 correspondingly. Our data suggest that significant dose reduction for optimized AAV-based vaccines is possible without sacrificing efficiency. In addition, we studied the role of conventional type 1 dendritic cells (cDC1) in optimized AAV-based immunization using a C57BL/6-Irf8em1Kmm (Irf8^*+*^32^−/−^) mouse model lacking cDC1. Interestingly, we found that cDC1 are not essential for conveying effector T cell responses to AAV-encoded tumor antigens.

## Graphical abstract

**Figure:**
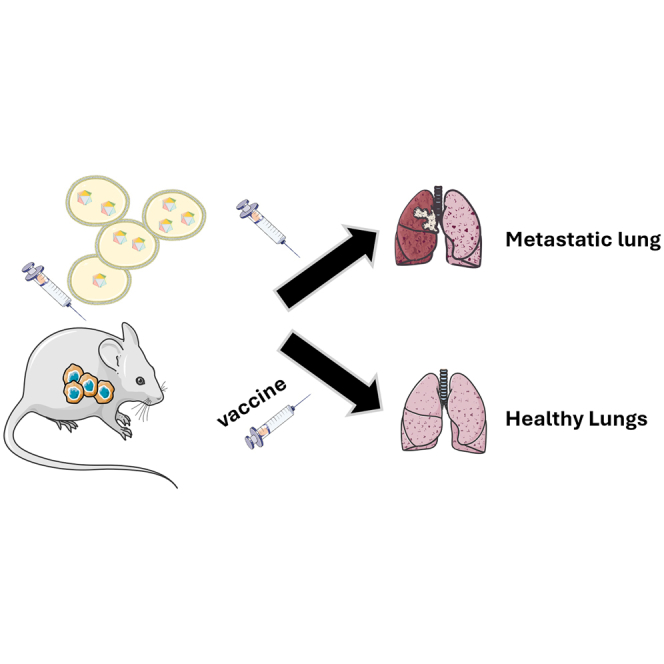

## Teaser

Aslanidi and colleagues developed and validated an effective vaccine delivery platform that naturally combined extracellular vesicles and adeno-associated virus vectors. The efficacy of vaccine was further tested on animal model that mimics human melanoma.

## Introduction

Adeno-associated viruses (AAVs) are a family of naturally occurring human and animal viruses with an extraordinary ability to deliver therapeutic modalities to host cells. AAV vectors are considered safe and efficient for clinical application, since there are no known diseases related to their infection and they have long-term episomal persistence in targeted tissues. Important advances in the gene therapy field have been achieved recently, with several AAV-based drugs approved by the FDA.^1^^,^^2^^,^^3^^,^^4^^,^^5^^,^^6^ The use of AAV vectors has also expanded to other areas of clinical applications. For example, they are used as vehicles to deliver neutralizing antibodies against infectious diseases such as the human immunodeficiency virus,^5^^,^^6^ and more recently as anti-COVID-19 vaccines,^7^ thus boosting opportunities for development of novel vaccines for cancer.^8^

The potential use of modified AAV vectors as anti-cancer vaccines has been documented in mouse models.^9^^,^^10^^,^^11^^,^^12^^,^^13^ Our previous results showed that AAV6-S663V carrying either ovalbumin (OVA)^14^ or tyrosinase-related protein 1 (TRP-1)^15^ genes, coupled with the MHC class I molecule-trafficking signals (opt-OVA and opt-TRP-1, respectively) markedly increased expansion of both CD8^+^ and CD4^+^ T cells. Intramuscular (i.m.) injection of these optimized vaccines (1 × 10^10^ vg per mouse) protected C57BL/6 mice from lung metastasis, and significantly delayed the growth of solid tumors in B16F10 and B16F10-OVA cell-based melanoma models.^14^^,^^15^

The general issue in viral-based cancer vaccines, including AAV, is immune response against viral capsids, which includes pre-existing neutralizing antibodies against AAV in humans,^16^ the innate immune system reaction against the capsids after virus administration, and the adaptive immune response activation toward cell infected with AAV.^17^^,^^18^ Several immunosuppressive strategies were developed for gene therapy of monogenic diseases,^19^^,^^20^^,^^21^ but cannot be implemented when AAV is used as a vaccine. Thus, a lower dose of AAV-based vaccine is necessary to avoid risk of initiation of the immune response toward capsids rather than encoded tumor antigen. We partially solved this problem by modifying surface-exposed residue S663V, which protects AAV from phosphorylation and sequential proteasomal degradation.^22^ This approach provided less material for presentation by antigen-presenting cells, and thus reduced anti-capsid immune response.^22^^,^^23^ In this study we proposed to use natural lipid membrane, extracellular vesicles (EVs), which increase transduction of AAV vectors and thus might shield the virus from immune attack.^24^

EVs are constitutively secreted by cells and can communicate with neighboring cells to deliver nucleic acids, proteins, lipids, or signaling molecules that can mediate physiologic or pathologic processes.^25^^,^^26^ In addition, EVs can readily diffuse through tissue and penetrate cellular barriers to exert their function.^27^^,^^28^Thus, EVs hold promise as novel delivery platforms.^29^^,^^30^ Several serotypes of AAV vectors were associated with EVs during standard production in HEK293 cells.^31^ Importantly, the EV-AAV complex has better transduction efficiency due to its high biocompatibility and does not require the specific receptor availability necessary for commonly used AAV vectors.^32^^,^^33^^,^^34^^,^^35^^,^^36^

In this study, we investigated whether we could increase the efficacy of cancer vaccines using a combination of capsid- and cargo-modified AAV6 vectors accumulated in EVs. First, we comprehensively characterized EV-AAV vesicles by their concentration, average diameter, size distribution, vesicle morphology, EV biomarkers, and AAV titers. Next, we showed that EV-AAV can generate a strong antigen-specific immune response with doses 10–100 times lower (depending on the antigen used) than previously published doses with common AAV-based vectors for immunomodulation. Significantly, interferon-γ (IFN-γ) produced CD8^+^ T cells, generated by mice vaccinated with either 10^8^ vg of EV-opt-OVA or 10^9^ vg/mice of EV-opt-TRP-1, and provided a similar protection against B16F10 cell-induced lung metastasis as 10^10^ vg of opt-OVA and opt-TRP-1, respectively.

Mechanisms of AAV-mediated immunity have been extensively studied, but interactions between AAV vectors and different subsets of dendritic cells (DCs) are not well understood. DCs are professional antigen-presenting cells with sentinel function. Conventional DCs (cDCs) play a vital role in initiating immune response toward antigens encoded by cancer vaccines and coordination between cDC1 and cDC2 is important for development of both CD8^+^ effector and helper CD4^+^ T cells.^14^^,^^37^^,^^38^ We showed that administration of capsid-optimized AAV6-S663V expressing green fluorescent protein into the biceps femoris muscle of mice directly targets local DCs (MHC-II^+^/CD11c^+^) that later migrated to draining lymph nodes,^14^ but exact subpopulations were not studied. In particular, cDCs were identified as important immune mediators of cytotoxic T lymphocytes responses^39^ for other cancer vaccines, and also involved in immune responses to AAV capsids.^40^ Thus, in the current study, we used a C57BL/6-Irf8em1Kmm (Irf8^+^32^−/−^) mouse model to dissect the role of cDC1 in immune response toward antigen encoded by our optimized AAV vaccine. These mice have a CRISPR-Cas9 421 bp deletion in the interferon regulatory factor 8 (Irf8) gene in the super-enhancer +32Kb. This deletion results in a complete depletion of all cDC1 lineage with no defects in other DC subpopulations.^41^^,^^42^ The opt-TRP-1 vaccine generated comparable numbers of IFN-γ-produced CD8^+^ T cells in both Irf8^+^32^−/−^ and wild-type C57BL/6 mice. Furthermore, Irf8^+^32^−/−^ mice vaccinated with opt-TRP-1 were protected from lung metastasis of B16F10 melanoma cells to a similar degree as C57BL/6 mice. Involvement of other subsets of DCs in optAAV vaccine function, such as plasmacytoid DCs, was not studied and required additional investigation.

In summary, we show the possibility of reducing AAV vaccine doses without reducing efficiency by employing EV-AAV symbiotic particles. Our data also suggest that cDC1 might be not essential for the development of tumor antigen-specific CD8^+^ T cells with killing capabilities.

## Results

### Characterization of the EV-AAV complex

**Figure 1:**
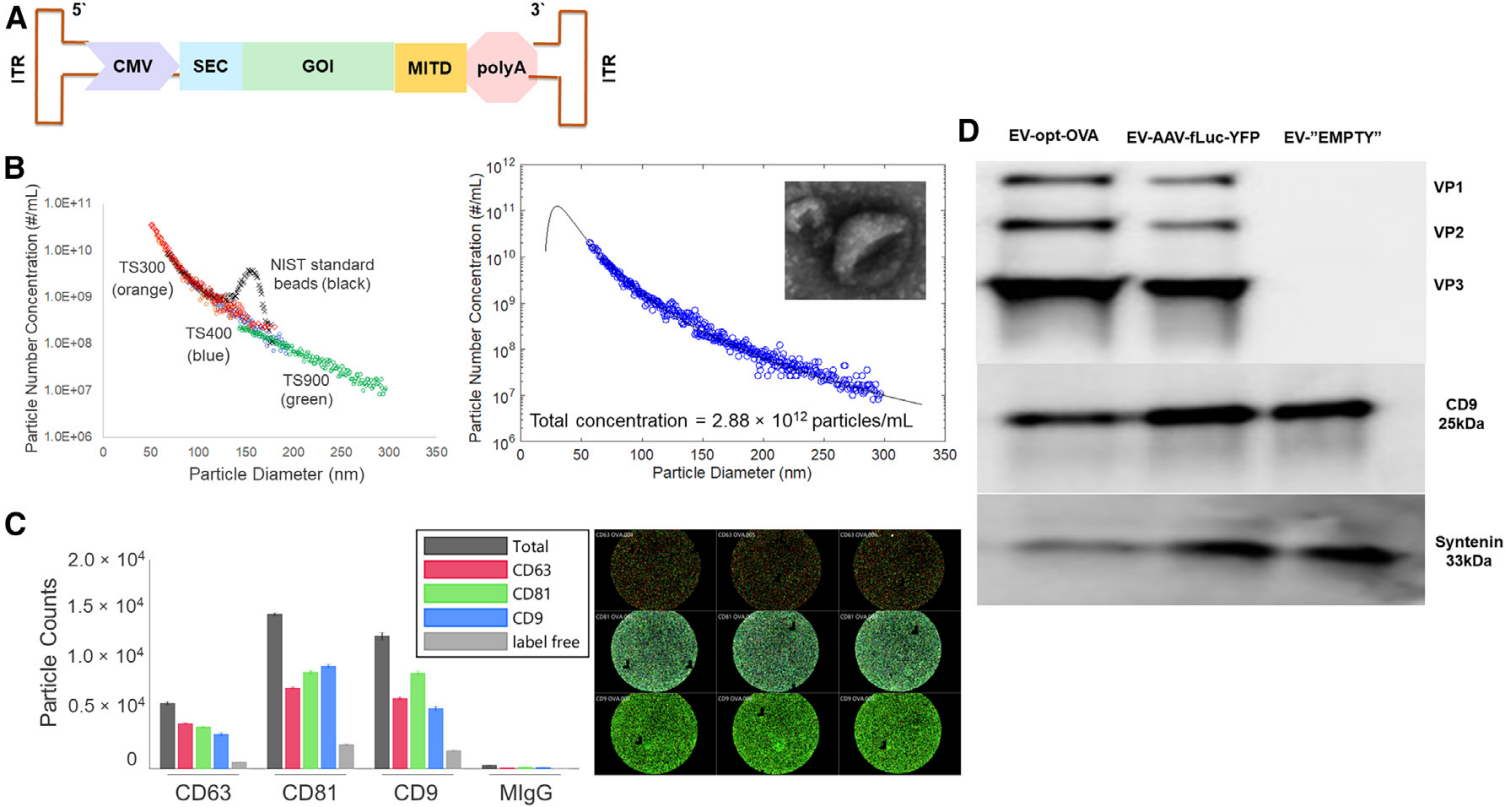
AAV expression cassette and EV-AAV characterization (A) Schematic outline of AAV expression cassette used in the study. (B) An example of particle size distribution measured by microfluidic resistive pulse sensing over a range of particle diameters from 50 nm to 1 μm. National Institute of Standards and Technology 150-nm-diameter standard beads were used for calibration. The model fit of the measured distribution shows scaling behavior (mode diameter ∼40 nm; scaling coefficient = 0.275) and gives a total particle concentration of 2.88 × 10^12^ particles/mL. Inset: representative TEM image of an EV-AAV6 particle (diameter ∼100 nm). (C) Particle counts obtained by SP-IRIS tetraspanin phenotyping using CD63, CD81, and CD9 capture. Fluorescence images of EVs co-expressing CD63, CD81, and CD9 captured on CD63 (top), CD81 (middle), and CD9 (bottom) spots. (C and D) Western blot analysis showing bands for VP1, 2, and 3 of the AAV6 capsid and for syntenin-1 and CD9, characteristic biomarkers for the exosome subpopulation of EVs. Equal titer of EVs were loaded.

First, we characterized EV-AAV preparation for size distribution, common EV markers, and viral titers. Total EV-AAV6 concentrations measured by microfluidic resistive pulse sensing^43^ were in the range of 10^11^–10^12^ particles/mL. Quantitative PCR of AAV titers on the viral genome showed a range of 3.89 × 10^10^–2.26 × 10^11^ vg/mL from several EV-AAV preparations, which is sufficient for use in mouse studies. EV-AAV6 size distributions measured by microfluidic resistive pulse sensing were unimodal and characteristically right-skewed to larger vesicle diameters (Figure 1B). A model fit of the measured distribution gave a peak at a vesicle diameter of ∼40 nm and showed scaling behavior with increasing diameter (scaling coefficient = 0.275). This scaling behavior and the scaling coefficient are characteristics of the asymmetry in exosome size distributions based on the established biogenesis pathway for exosome formation.^44^ No additional peaks in the distribution were observed for vesicle diameters up to 1 μm, the maximum detectable diameter of the measurement. For comparison, National Institute of Standards and Technology standard beads 150 nm in diameter used for calibration were readily detected at concentrations comparable with that of EV-AAV6 (Figure 1B). Transmission electron microscopy of EV-AAV6 particles showed spherical vesicles in the measured range of vesicle diameters with a distinctive cup-shaped morphology (Figure 1B inset).

Tetraspanin phenotyping using the single-particle interferometric reflectance imaging sensing (SP-IRIS) platform in fluorescence mode^43^^,^^44^ showed that EV-AAV6 vesicles co-express the common EV biomarkers—CD9, CD63, and CD81—although CD63 is less abundant relative to the other two markers (Figure 1C). Western blot analyses demonstrated the presence of both the exosome markers CD9 and syntenin-1^45^^,^^46^ and AAV capsid proteins in EV-AAV6 preparations (Figure 1D).

#### EV-opt-OVA-vaccinated mice develop protective immune responses at a low dose

**Figure 2:**
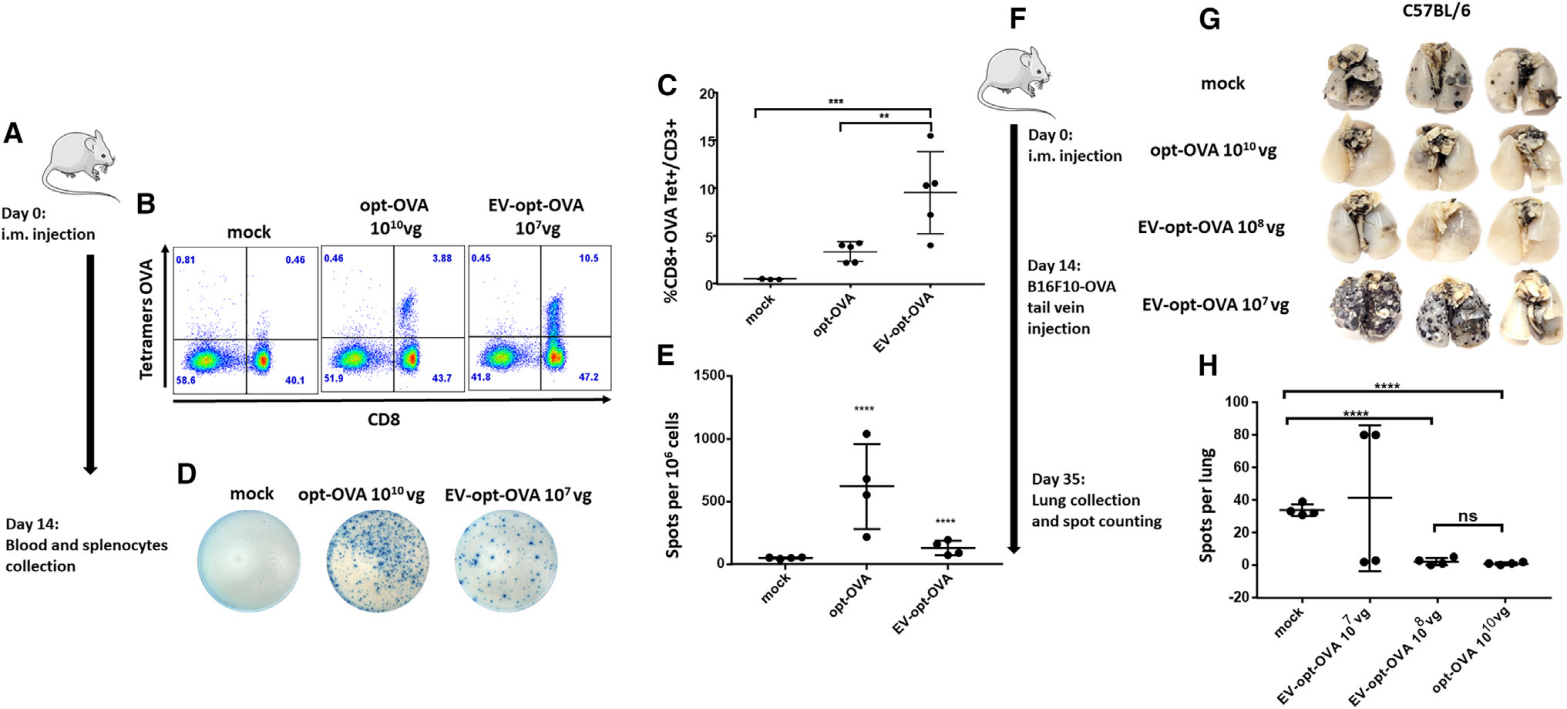
EV-opt-OVA generates protective immune response at lower doses than opt-OVA (A) Schematic representation of experiment timeline. (B) Representative flow analysis OVA-specific CD8^+^ T cells on mice PBMCs and (C) quantification of relative to the total number of CD8^+^ T cells on *n* = 4 mice. (D) IFN-γ release ELISPOT assay on spleens restimulated with OVA peptide and (E). Relative quantification and statistical analysis of IFN-γ positive spots per 10^6^ cells. (F) Schematic representation of experiment timeline. (G) Representative images of mice lungs were with B16F10 cells metastatic nodule quantification. Representative and (H) quantification based on *n* = 4 animals. ∗*p* = 0.01, ∗∗*p* = 0.001, ∗∗∗∗*p* < 0.0001.

We further investigated whether AAVs associated with EVs can increase the effectiveness of an optimized AAV vaccine and if vaccine dose can be further reduced but retain similar efficiency. First, we compared the generation of antigen-specific CD8^+^ T cells after i.m. vaccination of tumor-free mice with opt-OVA or EV-opt-OVA to identify the appropriate matching doses, i.e., the viral titer needed to induce comparable immune response. This was necessary because the two vaccine formulations are different and AAV viral titer is the only measurable standardized parameter. Flow cytometry analyses of blood with H-2K^b^-specific anti-OVA tetramers showed that a 10^7^ vg dose of EV-opt-OVA induced higher levels of anti-OVA-specific CD8^+^ T cells than a 10^10^ vg dose of opt-OVA (Figure 2A). On average, mice injected with EV-opt-OVA at 1 × 10^7^ vg had approximately 10% of specific CD8^+^ T cells in circulation 2 weeks post-immunization compared with approximately 4% of opt-OVA-treated mice in injected with 1 × 10^10^ vg (Figures 2B and 2C). The functional activity of generated OVA-specific CD8+ T cells was estimated as INF-γ release by splenocytes isolated from mice 2 weeks post-immunization and stimulated with OVA MHC class I immunodominant peptide, and measured by enzyme-linked immunospot (ELISPOT) assays. Mice injected with 1 × 10^7^ of EV-opt-OVA had fewer specific IFN-γ produced CD8^+^ T cells compared with the 1 × 10^10^ vg dose (Figures 2D and 2E). Since antigen-specific CD8^+^ T cells in the blood and spleen did not change proportionally between the EV-opt-OVA and opt-OVA groups, in the following experiments we used two doses of EV-opt-OVA vs. one dose of opt-OVA. We immunized C57BL/6 mice with 1 × 10^10^ vg of opt-OVA and with EV-opt-OVA at 1 × 10^7^ vg or 1 × 10^8^ vg and investigated whether the vaccine protected mice from lung metastasis of B16F10-OVA melanoma cells injected intravenously into the circulation.^27^ EV-opt-OVA at 1 × 10^8^ vg exhibited the same level of protection against metastatic tumors as opt-OVA at 1 × 10^10^ vg; EV-opt-OVA at 10^7^ vg was not significantly different than mock-treated controls (Figures 2F–2H). Based on these results, we concluded that IFN-γ ELISPOT assays of splenocytes better reflect the functional activity of antigen-specific T cells rather than the numbers of tetramer-positive T cells in the blood. Hence, the vg titers of EV-opt-OVA and opt-OVA given to mice in experiments described below were matched based on ELISPOT data.

#### Vaccination with EV-opt-TRP-1 requires a 10 times lower dose than opt-TRP-1

**Figure 3:**
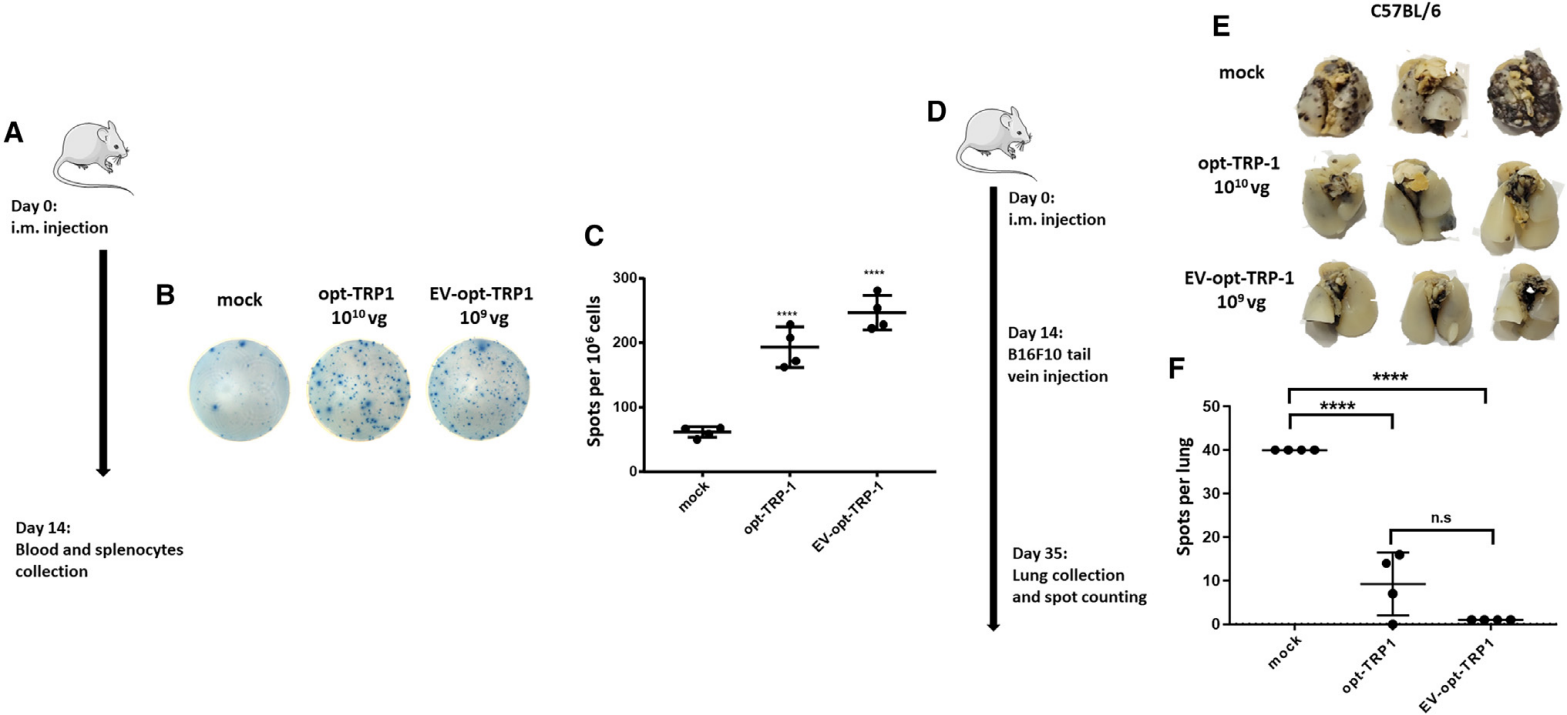
EV-opt-TRP-1 stimulates a protective immune response at lower doses compared with opt-TRP-1 (A) Schematic representation of experimental timeline. (B) Representative images of IFN-γ ELISPOT assay wells conducted on TRP-1 peptide restimulated splenocytes. (C) Quantification analysis of IFN-γ positive spots on *n* = 4 mice. (D) Timeline of B16F10 melanoma metastatic model experiment. (E) Representative images of C57BL6 mice lungs with B16B10 cell nodules. (F) Quantification of lungs nodules on *n* = 4 mice. ∗*p* = 0.01, ∗∗*p* < 0.001, ∗∗∗*p* < 0.005, ∗∗∗∗*p* < 0.0001.

OVA is a highly immunogenic antigen commonly used to study immune modulation, but it might not accurately represent the outcomes for vaccines against low immunogenic self-antigens. We recently showed that AAV vaccines carrying tumor antigens such as glycoprotein 100, tyrosinase, and TRP-1 protected C57BL/6 mice from B16F10 tumors to different extents.^15^ opt-TRP-1 vaccine was the most efficient and we chose it to produce EV-AAV to explore the possibility of reducing the therapeutic dose of the vaccine. The immunogenic capability of EV-opt-TRP-1 to elicit a CD8^+^ T cell response was analyzed in splenocytes after immunization with 1 × 10^9^ vg, and was similar to that of opt-TRP-1 at 1 × 10^10^ vg (Figures 3A–3C). Consequently, we used these matching doses to test protection against lung metastasis, as described above for OVA (Figure 3D). Mice injected with both opt-TRP1 and EV-opt-TRP-1 displayed significant reductions in lung nodules compared with mock-treated mice (Figures 3E and 3F).

#### Characterization of cDC subpopulations activated upon AAV administration

**Figure 4:**
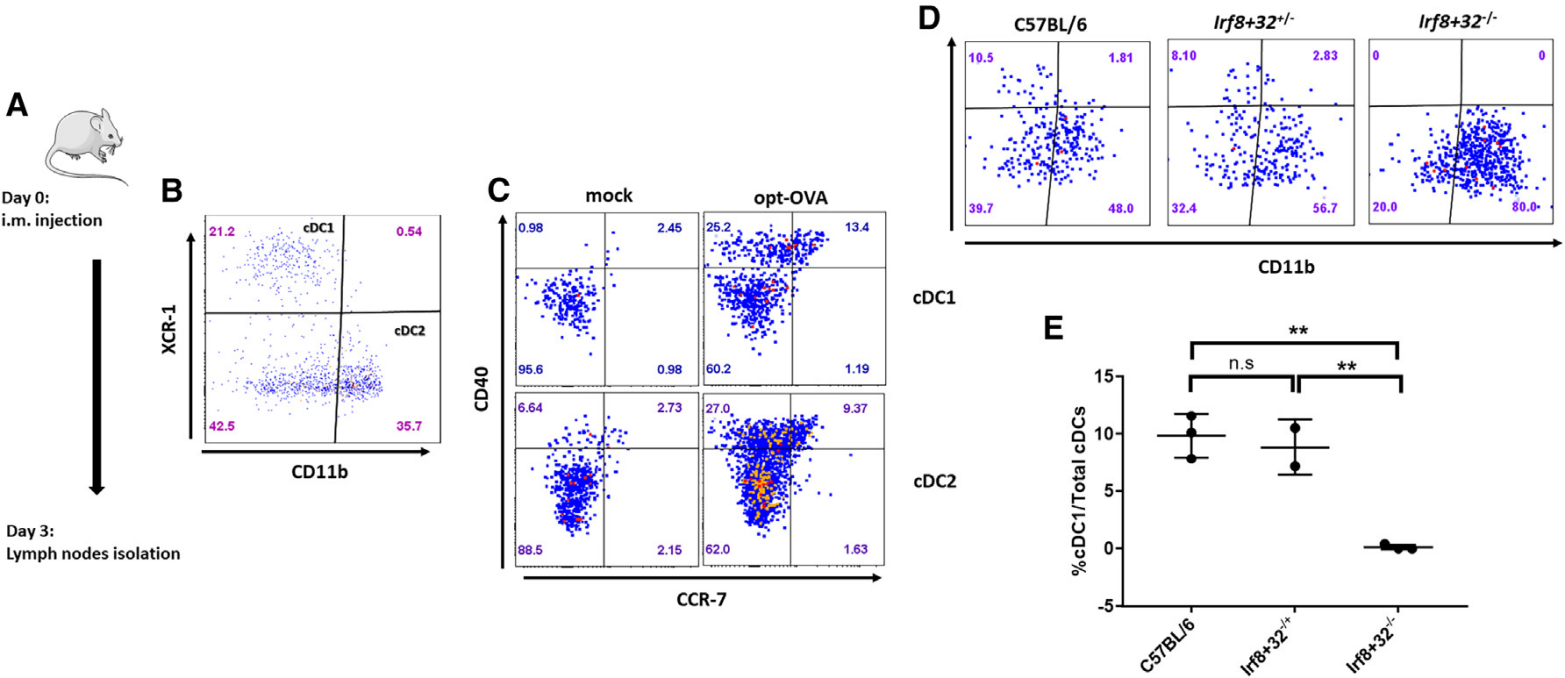
Analysis of cDC1 and cDC2 populations in wild-type C57BL/6 and Irf8^+^32^−/−^ (A) Schematic representation of experimental timeline. (B and C) Flow cytometry analysis of lymph nodes isolated from C57BL/6 mice injected with opt-OVA. Subpopulation of dendritic cells identified as cDC1 (CD11c^+^/MHC-II^+^/PDCA^−^/XCR-1^+^/CD11b^−^) and cDC2 (CD11c^+^/MHC-II^+^/PDCA^−^/XCR-1^–^/CD11b^+^). Activation of cDC1 and cDC2 define as upregulation of we used CD40^+^ and CCR7^+^ markers. (D) Comparison analysis of cDC1 and cDC2 subpopulations in Irf8^+^32^−/−^ vs. C57BL/6 and Irf8^+^32^+/−^ mice. cDC1 (CD11c^+^/MHC-II^+high^/XCR-1^+^/CD11b^−^) and cDC2 (CD11c^+^/MHC-II^+high^/XCR-1^–^/CD11b^+^). (E) Quantitative analysis of cDC1 subpopulation in Irf8^+^32^−/−^ vs. C57BL/6 and Irf8^+^32^+/−^ on *n* = 3 mice. ∗*p* = 0.01, ∗∗*p* = 0.001.

To investigate the immune mechanisms that AAVs trigger upon i.m. injection, we characterized different subpopulations of DCs in drained (inguinal) lymph nodes of mice. Our previous results suggested that optimized AAV6 vectors infect MHC-II^+^/CD11c^+^ DCs at the site of injection, which then migrate to lymph nodes within 48–72 h.^12^ To further explore this immune reaction, we determined which subpopulations of conventional DCs respond to AAV-mediated immunization. C57BL/6 mice were injected i.m. with opt-OVA (Figure 4A) and different populations of cDCs from drained lymph nodes were characterized by flow cytometry. We defined cDC subpopulations as cDC1 (CD11c^+^/MHC-II^+^/PDCA^−^/XCR-1^+^/CD11b^−^) and cDC2 (CD11c^+^/MHC-II^+^/PDCA^−^/XCR-1^–^/CD11b+). Upon opt-OVA injection, approximately 13% of cDC1 cells and 9% of cDC2 cells became activated based on CD40^+^/CCR7^+^ markers, compared with less than 3% of cDC1 and cDC2 cells in mice injected with phosphate-buffered saline (controls) (Figures 4B and 4C).

To further investigate the role of subsets of cDC in host immune response to AAV vaccines, we characterized the same cDC populations in Irf8^+^32^−/−^ mice.^25^ We confirmed that Irf8^+^32^−/−^ mice lacked CD11c^+^/XCR-1^+^ cDC1 in lymph nodes compared with controls such as heterozygous Irf8^+^32^+/−^ littermates and the C5BL/6 background strain and, hence, are suitable to dissect the role of cDC1 in immune response induced by our AAV vaccine (Figures 4D and 4E).

#### Mice lacking cDC1 develop specific CD8^+^ T cells against TRP-1 tumor antigen upon opt-TRP-1 vaccination

**Figure 5:**
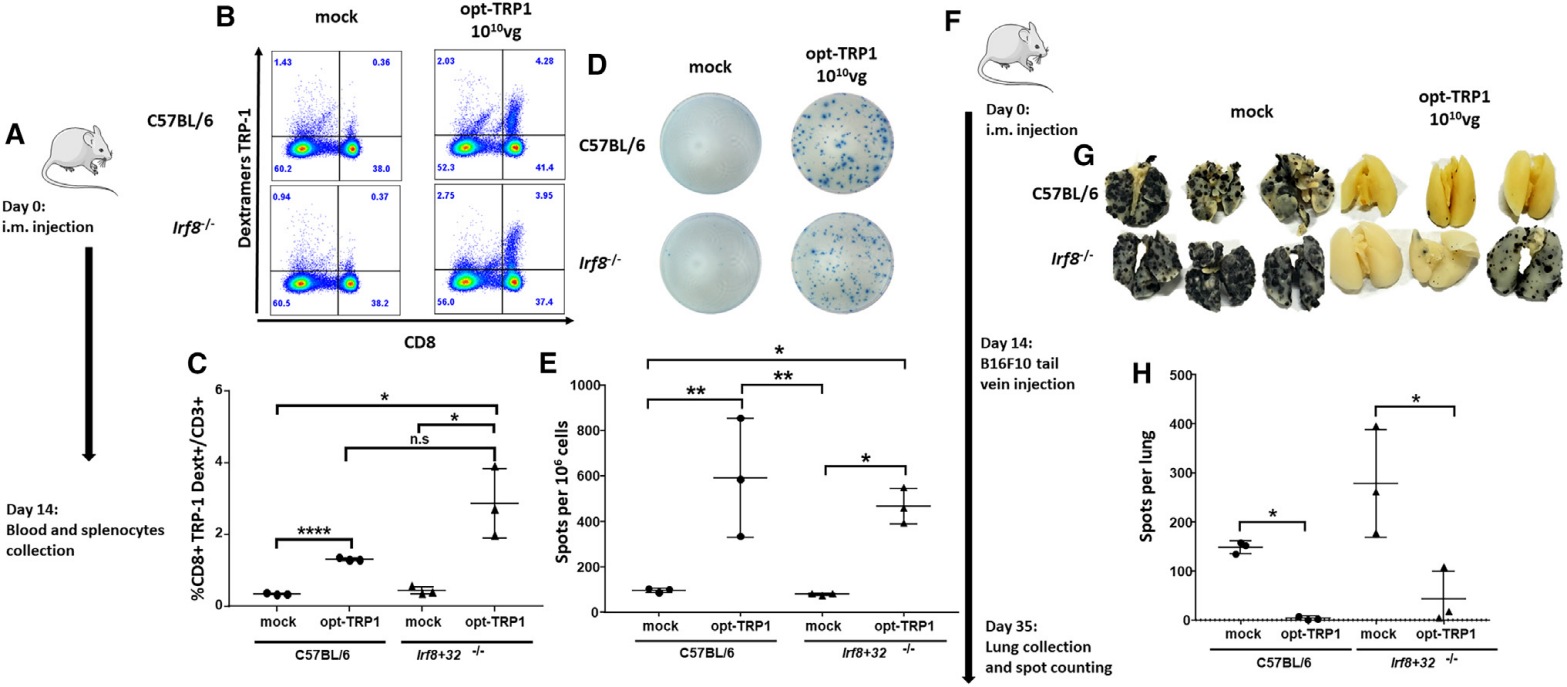
*Irf8+32*^−/−^ mice vaccinated with opt-TRP-1 develop specific CD8+ T cells against TRP-1 antigen with protective capabilities against metastatic spread in lungs (A) Schematic representation of experimental timeline and vector design. (B) Representative flow cytometry analysis TRP-1-specific CD8^+^ T cells extracted from opt-TRP-1-vaccinated Irf8^+^32^−/−^ and C57BL/6 mice and (C) quantification of *n* = 3 mice (Student’s t test). (D) Representative image of IFN-γ ELISPOT analysis on splenocytes extracted from opt-TRP-1-vaccinated Irf8^+^32^−/−^ and (E) C57BL/6 mice and quantification of *n* = 3 (multivariant ANOVA). (F) Schematic representation of experiment timeline. (G) Representative images of mice lungs with B16F10 cells metastatic nodules and (H) quantification of *n* = 3 mice (multivariant ANOVA). ∗*p* = 0.01, ∗∗*p* = 0.001.

We previously demonstrated that C57BL/6 mice injected with opt-TRP-1 developed antigen-specific cytotoxic CD8^+^ T cells and were protected against metastasis of B16F10 melanoma cells to the lungs compared with mock-injected mice.^12^ We questioned whether the lack of CD11c^+^/XCR-1^+^ cDC1 would abolish antigen-specific CD8^+^ T cell generation and consequent B16F10 clearance of melanoma cells metastatic to the lungs. C57BL/6 and *Irf8*^*+*^*32*^*−/−*^ mice were vaccinated with opt-TRP-1 (10^10^ vg) via i.m. injection. Peripheral blood mononuclear cells and splenocytes were analyzed 2 weeks after vaccination by flow cytometry and ELISPOT assays, respectively (Figure 5A). Both C57BL/6 and *Irf8*^*+*^*32*^*−/−*^ mice injected with opt-TRP-1 showed an increase in CD8^+^/TRP-1 dextramer+ T cells compared with controls (Figures 5B and 5C). ELISPOT assays confirmed that both C57BL/6 and *Irf8*^*+*^*32*^*−/−*^ mice vaccinated with opt-TRP-1 developed significantly more IFN-γ-produced T cells compared with mock-injected mice (Figures 5D and 5E).

Finally, we evaluated the functional efficiency of the opt-TRP-1 vaccine against B16F10 cells metastatic to the lungs in the absence of cDC1. C57BL/6 and *Irf8*^*+*^*32*^*−/−*^ mice were each vaccinated with 10^10^ vg of opt-TRP-1, and 2 weeks later received B16F10 melanoma cells through a tail vein injection as described previously^26^ (Figure 5F). Both groups vaccinated with opt-TRP-1 were protected against lung tumors compared with mock-treated mice (Figures 5G and 5H). The data suggest that the development of effector T cells induced by the opt-TRP-1 vaccine does not require cDC1 populations, at least in this mouse model.

## Discussion

AAV-based preventive or therapeutic immunization for infectious diseases^47^ and cancers^48^^,^^49^ has shown potential in animal models and early clinical trials with several important benefits besides safety and efficacy. For example, a considerable advantage of an AAV-based anti-COVID-19 vaccine is greater thermal stability and long-term viability compared with other vaccines that require very complex cold chain storage conditions.^7^ Another significant advantage is high compatibility with common cancer treatments such as radiotherapy^50^ or anti-PD-1 therapy.^14^ Importantly, the AAV vector doses used for vaccination studies and required for therapeutic benefits are significantly lower than doses used in gene therapy studies for inherited diseases, which can range from 1 × 10^11^ to 1 × 10^13^ vg per mouse.

The reduced AAV dose when used as a vaccine is beneficial for both production cost and safety requirements.^51^ To achieve desirable dose reduction without reducing therapeutic efficacy, several strategies were applied by our and other groups. We recently showed that an optimized AAV vaccine generated effective CD4^+^ and CD8^+^ T cell-driven anti-tumor immune responses at a dose of 1 × 10^10^ vg/mouse.^52^ We achieved these results with a rationally altered serine (S) to valine (V) residue at the position 663 and fused encoded antigen with MHC class I molecule trafficking signals.^14^^,^^15^^,^^22^ Another group described an innovative AAV modification with MHC class I OVA epitopes incorporated into the capsid. This modification improved host immune reaction and therefore reduced effective doses needed to achieve strong CD8^+^ T cell response, down to a dose of 1 × 10^9^ vg per mouse.^35^

The recent discovery of the possible production of EVs with AAV vector accumulated inside them opened an exciting approach for AAV delivery in general and particularly for vaccine design.^53^^,^^54^^,^^55^ EVs are considered immunomodulatory agents, since they can trigger either immune tolerance or immune response depending on the origins. For example, tumor-associated EVs are linked to inactivation of T cell response^56^ or suppression of natural killer cell function.^57^ Another way that EVs might avoid immune surveillance is to inactivate antigen-presenting cells such as DCs, particularly by manipulating the ability of monocytes to differentiate toward DCs or impair DC maturation.^58^^,^^59^ On the other hand, EVs derived from DCs inhibit tumor growth by activating IFN-γ producer CD8^+^ T cells, increasing IL-2 expression and inhibiting regulatory T cells.^60^ Furthermore, lipid-based adjuvants can increase therapeutic activity of a vaccine^61^^,^^62^^,^^63^ and EVs can serve as natural enhancers of vaccine-mediated immune responses.

We found that EV-opt-OVA and EV-opt-TRP-1 induced a robust CD8^+^ T cell immune response at a dose of 1 × 10^8^ or 1 × 10^9^ vg per mouse—100 and 10 times lower than our previously published^14^^,^^15^ effective doses of AAV, respectively. These differences in dose reduction suggest the advantages of using strong antigens such as OVA compared with self-antigens such as TRP-1 and highlight the necessity of discovering stronger endogenous immunologic targets.

An additional benefit of using EV-AAVs beside increased transduction efficiency is possible protection from the host immune system, particularly pre-existing or therapy-induced neutralizing antibodies against AAV vectors^64^^,^^65^ with seroprevalence among human populations reaching up to 40%–80% depending the population and serotype tested.^16^^,^^66^ The presence of these antibodies significantly reduces the number of eligible patients for AAV-based therapies and precludes vector re-administration. Thus, the use of EV-AAV could be beneficial if AAV re-administration is needed to boost therapeutic action of a cancer vaccine.^67^

Importantly, the production of EV-AAVs is a one-step process that makes it considerably more effective than commonly used electroporation for RNA or DNA therapeutics. Also, production of EV-AAV is straightforward, since conditioned cell culture medium is used for purification, and requires fewer cells to accumulate a similar AAV titer. However, better quality control is needed to effectively produce high amounts of EV-AAVs for clinical applications.^68^

We also studied the mechanism of immune stimulation in our optimized AAV vaccine, specifically the role of cDC subsets in stimulating antigen-specific CD8^+^ T cell proliferation. AAV6 vectors—unlike, for example, AAV8 vectors^69^—effectively transduce DCs both *in vitro* and *in vivo*.^14^^,^^22^^,^^70^ Previous studies indicate that cross-priming of capsid-specific CD8^+^ T cell activation requires both cDCs and plasmacytoid DCs,^71^ and depends on XCR-1^+^ cDC1. The conditional depletion of cDCs was related to a lack of anti-capsid CD8^+^ T cell activation upon AAV administration.^71^ In addition, impaired cDC1 function by deletion of either MHC class II or CD40 prevented rejection in a tumor-OVA model with immunization based on soluble antigen.^72^

We showed that opt-OVA activates both XCR-1^+^/CD11b^−^ cDC1 and XCR-1^–^/CD11b^+^ cDC2 during i.m. immunization, which can be found in lymph nodes 72 h after vaccine administration. To identify the contribution of cDC subsets involved in AAV-stimulated antigen-specific expansion to CD8^+^ T cells, we used *Irf8*^*+*^*32*^*−/−*^ mice with selectively depleted XCR-1^+^ cDC1.^41^^,^^42^ Absence of cDC1 in mice did not compromise generation of TRP-1-specific CD8^+^ T cells. Moreover, *Irf8*^*+*^*32*^*−/−*^ mice were protected against B16F10 tumor metastasis in the lungs, similar to C57BL/6 mice. Although previous studies indicated that cDC1 are important for cross-priming CD8^+^ T cells,^71^ we demonstrated that they are not essential to elicit effector T cell response to optimized AAV6 vaccine-encoded tumor antigens. Also, DCs are highly adaptable and readily alter their function in a condition-specific manner to support the appropriate T cell response. cDC2, generally responsible for CD4^+^ T cell stimulation, can also cross-present antigen to CD8^+^ T cells,^73^^,^^74^ which might occur in our studies with Irf8^+^32^−/−^ mice. Additional studies are needed to elucidate the role of cDC2 in AAV-mediated antigen-specific immunity or identification of alternative antigen-presenting cells involved in AAV vaccine function, for example, pDC^75^^,^^76^ or CD169^+^ macrophages.^77^ Also, further validation of vaccination approach with optAAV or EV-optAAV need to be conducted to evaluate other important modalities of immune response, such as humoral responses and activation of T memory cells, and response to the tumor cells re-challenge.

Our findings justify use of alternative routes of AAV vaccine administration such as intradermal and intralymph nodes since cDC2 is abundantly present in those sites. Indeed, several attempts have been made to improve intradermal administration of common vaccines such as influenza,^78^ hepatitis B,^79^ or poliovirus,^80^ to reduce the dose and sparing the efficacy of the vaccine^81^ and DNA-based melanoma vaccine.^82^ These results suggest a promising strategy to change the route of administration for AAV-based vaccination.

Based on our data, we propose that relatively low genomic titers of AAV-based vaccines can achieve strong therapeutic effects by capitalizing on the natural ability of AAV vectors to be loaded into EVs in producer cell lines. Second, we revealed, in part, a mechanism of function for our optimized AAV vaccines expressing self-tumor antigens that we showed can target DCs directly, and do not require a cDC1 subset to stimulate antigen-specific immune response, unlike the anti-AAV capsid immune response that depended on cDC1. Our data could further help advance the development of anti-cancer vaccines based on available immunogenic targets.

## Materials and methods

### Animals

C57BL/6 and breeding pairs of C57BL/6-Irf8em1Kmm (Irf8^+^32^−/−^) mice were obtained from Jackson Laboratory (Bar Harbor, ME). In all experiments, both male and female 6- to 10-week-old mice were used. All experiments with mice were performed according to the principles of the National Research Council’s Guide for the Care and Use of Laboratory Animals and with approval by the University of Minnesota Institutional Animal Care and Use Committee.

### Vectors

The OVA antigen or truncated mouse TRP-1 (257–506 amino acids), which include the immunodominant peptide^83^^,^^84^^,^^85^ with a single A463M mutation to increase immunogenicity, were coupled with MHC class I trafficking signals to improve the presentation of MHC class I and class II epitopes in DCs,^86^ as described previously.^14^ Both antigens were packaged in AAV serotype 6 containing a single mutation in the VP3 capsid protein at amino acid position 663 to substitute S with V.^22^ The same vectors that expressed firefly luciferases (AAV6-S663V-fluc) were used as non-specific controls (mock treatment). Vectors were packaged in HEK293 cells and purified by an iodixanol gradient followed by ion-exchange column purification.^14^^,^^22^ EV-AAVs were pelleted from approximately 100 mL of the conditioned medium by centrifugation at 100,000 × *g* for 2 h at 4^o^C. Pellets were then resuspended in 500 μL sterile phosphate-buffered saline and subsequently filtered by centrifugation through a 0.45-μm centrifuge filter (Spin-X, Thermo Fisher Scientific) at 10,000 × *g* for 15 min.^36^

### EV-AAV characterization

EV concentrations and size distributions were quantified by microfluidic resistive pulse sensing using an nCS1 nanoparticle analyzer (Spectradyne, Signal Hill, CA) with C300, C400, and C900 cartridges covering a size range of 50 nm to 1 μm as described in detail elsewhere.^43^ Imaging was carried out on a Phillips CM-120 transmission electron microscope operating at 80 kV with an AMT XR80 CCD (8 megapixels) as described in detail elsewhere.^87^ SP-IRIS in fluorescence mode was conducted for tetraspanin phenotyping of EVs and EV AAV6 using ExoView Human Tetraspanin chips (Unchained Labs, Pleasanton, CA) printed with anti-human CD81, CD63, and CD9 monoclonal antibodies, and anti-mouse IgG1 antibody to control for non-specific binding, as described in detail elsewhere.^43^ All chips were imaged in the ExoView scanner (Unchained Labs) by interferometric reflectance imaging and fluorescent detection. Data were analyzed using ExoView Analyzer 3.1 software (Unchained Labs).

Expression of viral capsid protein (VP) and the common EV markers CD9 and syntenin-1 was analyzed by western blotting on equal amounts of EV and EV-AAV.^36^ In brief, EV lysates were separated on 10% polyacrylamide/SDS gels and transferred to nitrocellulose membranes. Primary antibodies, anti-VP (VP1+VP2+VP3); clone B1 (mouse mAb 1:2,000), anti-CD9 clone TS9 (mouse mAb 1:1,000), and anti-syntein-1 clone 2B8 (mouse mAb 1:1,000) followed by secondary horseradish peroxidase-linked antibodies (1:1,000) (Cell Signaling Technology, Danvers, MA) were used to visualize protein expression. Precision Plus Kaleidoscope Prestained Protein Standards (Bio-Rad, Hercules, CA) were used to relate molecular weights of viral proteins.

### Cells and tissues

B16F10 mouse melanoma cells (ATCC) were maintained in Dulbecco’s modified essential medium supplemented with 10% fetal bovine serum (Gibco), 100 U/mL penicillin, and 100 μg/mL streptomycin, or with addition of 500 μg/mL G418 for B16F10-OVA.

Peripheral blood mononuclear cells were isolated from the blood collected by facial vein bleeding into EDTA tubes, and spleens or inguinal lymph nodes were homogenized by passing through a cell strainer, and cleaned from erythrocytes by adding red blood cell lysis buffer. Single-cell suspensions were resuspended in RPMI supplemented with 10% fetal bovine serum (Gibco), 100 U/mL penicillin, and 100 μg/mL streptomycin, or in flow staining buffer (1% fetal bovine serum in phosphate-buffered saline plus 0.025% sodium azide) depending on downstream applications.

### Lung tumor nodules

Mice were injected in the biceps femoris muscle with a single dose of 1 × 10^7^–1 × 10^10^ vg per mouse depending on AAV vaccine composition and encoded antigen. At 2 weeks post-vaccination, mice were injected with 4 × 10^5^ B16F10 cells or 8 × 10^5^ B16F10-OVA cells via the tail vein. Eighteen days after tumor challenge, the mice were euthanized, and the lungs were briefly rinsed with tap water to remove blood and bleached overnight in Fekete’s solution. The next day, black tumor nodules formed by B16F10 or B16F10-OVA cells were counted.^14^^,^^15^

### Flow cytometry and antibodies

Fluorophore-conjugated antibodies used were: CD45 (30-F11), CD3e (145-2C11), CD11c (N418), CD11b (M1/70), CD40 (3/23), CCR7 (4B12), XCR-1 (ZET), CD44 (IM7), MHC-II (I-A/I-E) (M5/114.15.2), CD8 (KT15) (Thermo Fisher Scientific), H-2Kb MHC class I OVA_257-264_ tetramers (SIINFEKL, MBL, International), and custom H-2K^b^ MHC class I TRP1_455-463_ dextramers (TAPDNLGYA, Immudex). Dead cells were excluded by positive staining with 7-AAD (BioLegend), and all data were gated on live and single cells (FSC-A vs. FSC-H). Data were acquired using a BD FACS LSR Fortessa or BD FACS Aria II instrument, and raw data were analyzed by FlowJo software (version 10.0.3).

### ELISPOT assays

IFN-γ production in response to restimulation with corresponding immunodominant peptides was assessed by ELISPOT assays (Cellular Technology, Shaker Heights, OH) and performed as described previously.^14^^,^^15^ In brief, splenocytes were seeded at 2 × 10^5^ cells per well and restimulated with MHC class I immunodominant peptide at a final concentration of 1 μg/mL. The peptides for H-2Db-restricted epitope mTRP1_455-463_ TAPDNLGYA (GenScript), and H-2Kb restricted epitope OVA_257-264_ SIINFEKL peptides (Anaspec) were used.^14^^,^^15^ Plates were cultured for 48 h and developed according to the manufacturer’s instructions. IFN-γ-secreting spots were counted with an ELISPOT reader. The results are presented as the number of spots per 1 × 10^6^ cells.

### Statistical analysis

All data were analyzed and plotted using GraphPad Prism software. Data are shown as the mean ± SEM. For all statistical analyses, unpaired t tests were used. Data were considered significant when *p* values were <0.05.

## Data and code availability

Data and materials described in this manuscript will be available upon request to the corresponding author.

## Acknowledgments

This project was supported by a Paint the Town Pink pilot grant (to G.A.), a Fifth District Eagles Cancer Telethon Postdoctoral Fellowship Award (to H.K.), and startup funds from the Hormel Institute (to G.A.).

## Author contributions

E.M., M.T., K.K., and G.A. developed the concept of the project and designed the experiments. E.M., M.T., O.F.D., C.C., H.K., K.K., and M.P. performed the experiments. E.M., M.T., and M.P. analyzed the data. E.M. and G.A. wrote the manuscript. M.T., O.F.D., C.C., H.K., K.K., and M.P. edited the manuscript.

## Declaration of interests

G.A. and K.K. have several issued or provisional patents related to AAV vectors that have been licensed to various gene therapy companies.
